# The social accountability of burn displays

**DOI:** 10.1177/14614456251363736

**Published:** 2025-11-15

**Authors:** Hanna Svensson, Sofian A. Bouaouina, Guillaume Gauthier

**Affiliations:** 1 University of Basel, Switzerland

**Keywords:** sensoriality, accountability, pain display, multimodality, response cries, retro-sequences

## Abstract

This study examines the social accountability of displaying to burn oneself during naturally occurring joint cooking activities. The sequential, multimodal analysis of responding actions to burn displays allows to discuss publicly available displays of perceptual experiences as related to social action. The study specifies three sequential trajectories following displays of heat-occasioned experiences, including (i) orienting to the unfortunate character of the event, (ii) orienting to issues of responsibility and (iii) questioning the validity of burn displays. The study shows that the question of what observable conduct is physiological and what is social is a members’ problem, including whether, how and to what extent the burn display is proportional to the object’s thermic features, and the sequential and moral implications that the burn display makes relevant. The study contributes to our understanding of retrosequences as a sequence organization that incorporates the relevance of sensorial practices in and for social interaction and of how an EMCA approach to the study of sociality can be particularly productive for further investigating the intricate relation between physiological experiences and the social accountability of action. The participants speak French, Swiss-German and German as first and second language.

## Introduction

**Image 1.**
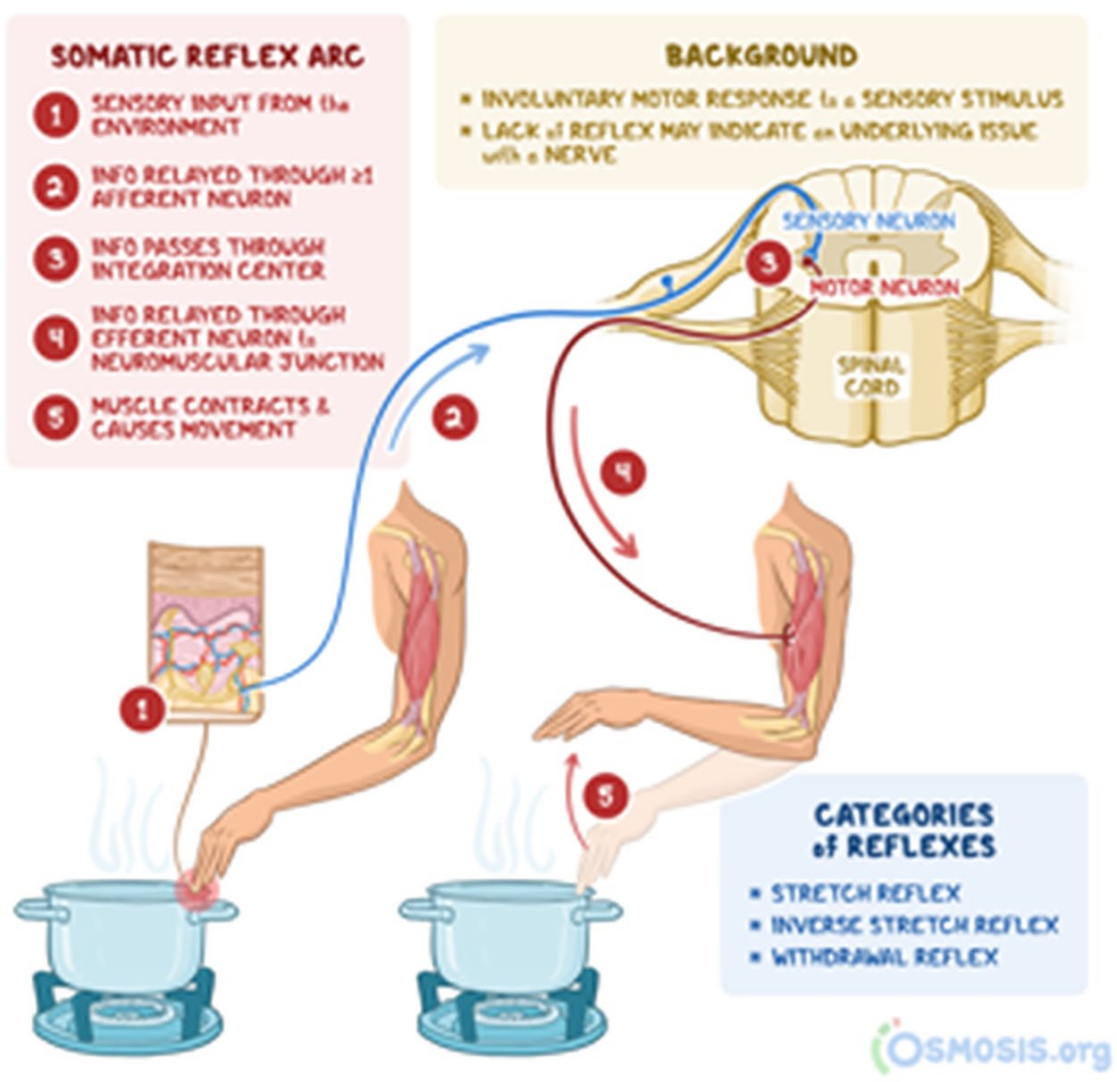
Visual representation of the somatic reflex arc. Source: https://www.osmosis.org/answers/somatic-reflex.

Bodily exposure to hot objects recurrently occasions conduct with characteristic physiological features, including retracting the body part from the heat source. In the natural sciences, such retractions are considered a basic withdrawal reflex related to the nervous system. Grossly put, when an external input that might cause tissue damage enters in contact with sensory neurons, this information passes through to motor neurons that cause muscle contraction and a withdrawing movement, essentially preventing serious harm to our bodies. Image 1 represents such a retraction as an isolated, strictly physiological, and automated ‘internal’ process.

However, actual occurrences of such incidents are embedded within situated activities, unfold over time and are not limited to a retraction of the forearm. An example of this is presented in Excerpt 1 below, drawn from video recordings of a cooking class, where Anna burns herself on the tap water as she washes her hands. Her classmate Lea is already by the sink. The multimodal transcripts draw on Jefferson (2004), ICOR Group (2007), and Mondada (2018) conventions.

Excerpt 1: SCHOOL_HausW_DEL_20210122_K2_PM_ILOT2_00.39.56

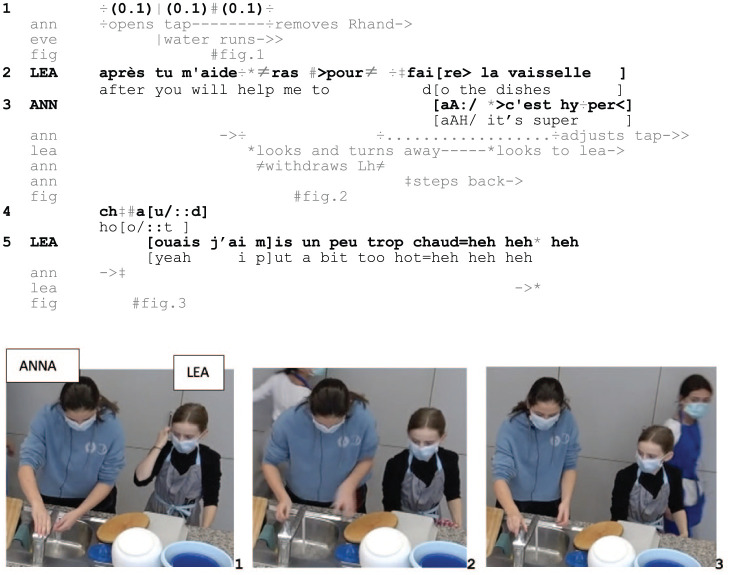

From a ‘scenic’ perspective (Garfinkel, 1963: 190), the intelligibility of Anna’s conduct as her burning herself lies in the specific, temporally unfolding *assemblage* of multiple embodied and vocal resources in a particular context. As Anna opens the tap and starts washing her hands (1–2, Fig. 1), Lea asks her for help with the dishes and projects to walk away, turning away from Anna (2). However, during Lea’s turn, Anna hastily retracts her hand from the water and steps backwards, thus suspending her course of manual action, and produces a loud and elongated vocalization, ‘aA:/’ (2–3, Fig. 2). In addition, and instead of responding to Lea’s request, she accounts for what she does as related to the water temperature (3–4). Lea orients to this conduct as making relevant an inspection of what occasioned it (Schegloff, 2007) and responds to it: Firstly, she reorients to Anna just after the vocalization (3, Fig. 3), allowing her to check the reason for the outcry and visually access her bodily withdrawal. Secondly, Lea first affiliates with Anna’s conduct through the *ouais*/‘yeah’ and then retrospectively takes responsibility for the event, treating the noticing as a complaint (cf. Pomerantz, 1978).

Lea’s responsive action ultimately establishes Anna’s conduct as a sensorial practice that is contingent on a heat-occasioned experience and made inspectable by virtue of her assemblage of (interactional) resources that render it publicly available and socially relevant to attend to. The embodied and verbal burn display (i) accountably interrupts Anna’s ongoing action of washing her hands, (ii) suspends the immediate interactional relevance of responding to Lea’s question, and (iii) projects the relevance of further action, which is established in Lea’s affiliative return.

In our data, we observe that participants in continuing states of incipient talk (Schegloff and Sacks, 1973) during joint cooking activities, who get in haptic contact with hot objects, recurrently assemble embodied and verbal resources into socially accountable heat-occasioned sensorial practices (Mondada, 2021) that participants treat as initiating retro-sequences (Schegloff, 2007). This raises critical questions concerning the public availability of physiological experiences and how they relate to social action. How do participants attend to conduct they treat as associated with sensorial, heat-occasioned experiences? What are members seen to be doing when they assemble and situate such conduct in this sequential environment? What is the *social accountability* of displaying to burn oneself?

In this study, we specify three sequential trajectories following displays of heat-occasioned experiences, including (i) orienting to the unfortunate character of the event, (ii) orienting to issues of responsibility and (iii) questioning the validity of burn displays. This will allow us to discuss the interactional relevancies projected by multimodally assembled burn displays in this environment and elaborate on the distinction between physiological and social conduct as being a members’ problem.

### Sensing as an interactional practice

Sensoriality has recently received increasing attention in ethnomethodological and conversation analytic (EMCA) (Garfinkel, 1967; Sacks, 1992; Sacks et al., 1974) research. In this study we are interested in the social accountability of displayed sensorial experiences that are occasioned by the participants’ haptic contact with hot objects, which we will treat as a form of touch.

As a sensorial practice, touch has mainly been examined in relation to the notion of ‘haptic sociality’ (Goodwin, 2017) and on bodies touching other bodies (Cekaite and Mondada, 2020; Nishizaka, 2020). This differs from touching objects in interaction, where the experience of materiality is distributed among bodies and their respective haptic accesses, thus requiring interactional resources to achieve intersubjectivity (Kreplak and Mondémé, 2014; Mondada et al., 2021). In our data, the participants regularly engage physically with objects that appear to be hot and make heat-occasioned experiences publicly available and interactionally relevant through the assemblage of embodied, vocal and verbal resources. The noticeability of heat-occasioned sensorial experiences *as a practical accomplishment* has, to our knowledge, not yet been discussed in the literature. Furthermore, the participants’ displayed, and locally established haptic experiences are exhibited and treated as being painful, which establishes their particular social accountability.

### Displaying pain in interaction

The social aspect of pain displays has been established in prior research. Goffman (1978) addresses pain displays as solitary but publicly accessible forms of expression in his work on ‘response cries’. Regarding the ‘pain cry’, Goffman (1978) notes that when it is produced because of experiencing pain and in the presence of witnesses, it implies that ‘our current, inner acutely-painful state is the business everyone should be hanging on’ (p. 804). In this way, he claims that a conduct that is occasioned by events that are seen as physiologically-laden and disjointed from the social interaction can be consequential for the interaction.

EMCA research has examined pain displays as social and public expressions of physiological experiences and as recurrent phenomena in medical physical examinations, where they are fundamental for many diagnostic courses of action (Heath, 1989; La and Weatherall, 2020; Weatherall et al., 2021) and used as resources for action (Heath, 1991; McArthur, 2021). McArthur (2021) argues that ‘[p]ain displays are *not* merely automatic reactions to internal experiences of pain. Yet, it is precisely our shared commonsense view of them as such that makes them available as resources for action in the first place’ (pp. 280–281). In line with Goffman’s observations, this research shows that displaying painful experiences that come across as uncontrollable make relevant to show emotion and affect, which, in turn, makes them accountable resources people can attend to.

Whereas most research on pain displays has examined interactions where their occurrence is expectably tied to the procedure’s interactional organization, Weatherall et al. (2021) examine the interactional relevance of unexpected displays of pain in doctor-patient interaction. Our study contributes to this body of research by focusing on instances where displayed painful experiences are produced and treated as being unexpected and contingent on material conditions. We are interested in how displays of heat-occasioned experiences, as vernacular sensorial interactional practices, can be progressively established as sequentially implicative during commonplace activities. We will show how displaying to burn oneself can set up the relevance of showing affiliation and sympathy, but also that it invokes issues of responsibility and that the validity of these displays can be questioned and/or contested. This, in turn, ultimately hinges on the intersubjectivity of sensorial experiences as practical, sequentially organized interactional achievements.

### Accountability and sequentiality in interaction

Within EMCA, we assume that compositions of embodied resources are produced as, and seen to be produced as, ‘visibly-rational-and-reportable-for-all-practical-purposes, i.e., “accountable,” as organizations of commonplace everyday activities’ (Garfinkel, 1967: vii). The accountable character of social action thus concerns its intelligibility as intrinsic to its social relevance (Garfinkel, 1967; Garfinkel and Sacks, 1970; Robinson, 2016). Both visible and audible embodied resources are well established in the literature as fundamental for achieving the accountability of social action. Indeed, conducts which have been considered as merely physiological, such as laughing and breathing, can be constitutive features of action formation (e.g. Jefferson, 1979, 1984; Keevallik and Ogden, 2020; Mondada, 2020).

From an EMCA perspective, the issue of action formation is embedded in a members’ question as ‘why that now?’, as participants to interaction continuously inspect their respective conduct for what it is doing and what it makes relevant to do next (Schegloff and Sacks, 1973: 299). The situated accomplishment of intersubjectivity relies on the public availability of action recognition as a members’ problem, which is eventually established in third position (Moerman and Sacks, 1988; Sacks et al., 1974: 728–729; Schegloff, 1992).

At the heart of this lies *sequentiality*, a notion that addresses the temporal relationship between events that are embedded in and make up to unfolding courses of action in and as sequences (Sacks, 1987; Schegloff, 2007). EMCA research has pervasively focused on prospective sequences as a prevalent organization of action, notably the adjacency pair (Schegloff, 2007; Schegloff and Sacks, 1973). There are however other organizations of action, including *retro-sequences*, that operate *retrospectively*, as they are initiated subsequently to what occasioned them in the prior interactional or material environment (Schegloff, 2007: 217).

### Retro-sequences and noticings

Although scarce, there is increasing research on actions with a retrospectively oriented organization, which has principally focused on lexicalized noticings of material features in the immediate environment (Song and Licoppe, 2024; Stukenbrock, 2023) and noticings of features of ongoing courses of action within instruction activities (Lindwall and Mondada, 2025). Vatanen (2023), however, discusses cases where participants to choir rehearsals make *embodied* noticings of both ‘self’s’ and ‘other’s’ emerging problems with the singing.

Schegloff (2007) describes ‘retro-sequences’ as linked to the larger action class of noticings: ‘[d]oing a noticing [. . .] works by mobilizing attention on the features which it formulates or registers, but it treats *them* as its source, while projecting the relevance of some further action in response to the act of noticing’ (p. 219; see also Sacks, 1992). These two aspects, (a) formulating or registering contextual features as their source, and (b) projecting the relevance for further action, are constitutive of retro-sequences. In our data, participants recurrently make publicly available their sensorial experience, mobilizing collective attention to what happened, and we can observe that these moments are systematically oriented to as making relevant subsequent action.

The formal features of burn displays are akin to what Goffman labelled as ‘response cries’. In line with Schegloff’s (2007) proposition that laughter and crying ‘[. . .] and possibly all of those expressions which Goffman (1978) termed “response cries” [. . .]’ (p. 219) are examples of noticings – that is, of action organization revolving around a source-outcome relationship.

Schegloff insists on the distinction between noticing as a perceptual/cognitive event and noticing as an interactional event, which makes a perceptual event socially available (Schegloff, 2007: 87 fn. 17) and which can be implemented verbally or by other embodied means. This distinction allows to point at how, from a members’ perspective, noticings are essentially bound to sensorial matters – whether in the wider scope of noticings as instantiations of retro-sequences or in the narrower, more vernacular act of noticing things. In this study, we address how participants make sensorial experiences *interactionally* noticeable, occasioning further action by co-participants, who exhibit their understanding that the pain display is contingent on the burn. In this way, we contribute to research on pain displays and response cries as sequentially organized social phenomena.

## Data

This paper draws on a corpus of video recordings carried out between 2019 and 2022 within the SNSF-funded project *From multimodality to multisensoriality*. It amounts to about 100 hours of naturally occurring interactions with and around food in a variety of settings, where people engage in various cooking activities. The videographic work includes documentation of cooking-classes in middle school, a food hackathon, a scout camp, a pop-up amateur restaurant, and various dinner preparations with friends and family. Recordings were designed to document the participants’ sensorial experiences. The languages used in the instances discussed in this paper include French, German as first and second language and Swiss-German. All participants (and their legal guardians) have given their informed consent about the recordings and their scientific use. The participants’ names have been pseudonymized.

## Methodological considerations

Cooking is a perspicuous setting for looking at the public availability and interactional relevance of haptic, sensorial phenomena given that the intersubjective achievement of sensorial experiences recurrently emerges as constitutive of the activity. Situated within EMCA, this research aims to reconstruct the participants’ displayed understanding of the unfolding interaction and to specify and explain the varying and recurring features of burn displays.

The study is based on 38 instances where participants make noticeable heat-related experiences during cooking activities. Following a case-by-case analysis, we consider the instances discussed in this paper to be representative of the proposed analytic subsections (cf. Schegloff, 1996). The English translations are idiomatic and aim to stay faithful to the original utterance while ensuring the readability of the unfolding interaction. When grammatical and other linguistic resources are consequential for the analysis, they are topicalized and explicated.

## Results

In the introduction, we observed that displaying to burn oneself projects the relevance of some further action and is treated as initiating a retro-sequence. In what follows, we elaborate on the sequential trajectories that reflexively establish the social accountability of making such sensorial experiences noticeable. We first discuss instances where co-participants attend to burn displays by orienting to the unfortunate nature of the event. We then discuss cases where participants orient to issues of responsibility in response to burn displays and in the subsequent section cases where participants question the validity of such displays. Finally, we discuss a case where the non-attendance to a burn display is treated as noticeably absent, which indicates the socio-interactional implications tied to pain displays.

### Orienting to the unfortunate character of burn displays

In this section, we show that co-participants can attend to burn displays by orienting to the unfortunate character of the event and by construing it as a mishap (see Pomerantz, 1978). In Excerpt 2, we join the cooking activity as Nina is pouring out pasta water with tagliatelle into a colander and burns herself on the steam from the boiling water. Her classmate Dario is standing on the other side of the kitchen island.

Excerpt 2: SCHOOL_HausW_SIS_20190402_I3_02.21.26

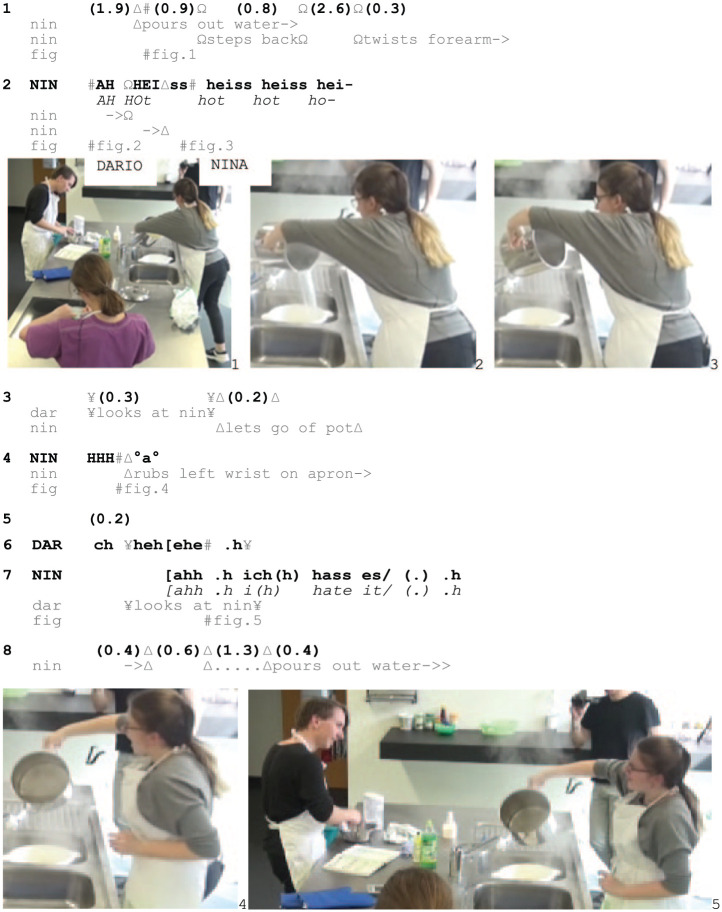

Shortly after starting to pour out the pasta water (1, Fig. 1) Nina steps backwards and twists her forearms (1–2, Fig. 2–3), creating as much distance between her body and the water as possible without letting go of the pot. After the outcry ‘AH’ Nina verbalizes the progressively aggravated problem of pain by repeating the lexical account *heiss*/‘hot’ (2), then stops pouring out water, retracts one hand from the pot and rubs her wrist on her apron while exhaling loudly (3–4, Fig. 4).

Although Dario might perceive Nina’s conduct from the beginning, he visibly looks at her only following her audible conduct (2), treating what she is doing as socially relevant. In response to her additional outcry (4), Dario laughs while looking at her again (6, Fig. 5), attending to her burn display as sequentially implicative, initiating a retro-sequence (Schegloff, 2007). Dario’s laughter does the work of acknowledging the unfortunate character of the burn and Nina’s conduct as invoking trouble (Haakana, 2001; Jefferson, 1984), while not engaging further with the event (see Holt, 2012). Nina, on the other hand, pursues the topic by explicating that she ‘hates it’ (7, Fig. 5), escalating the complainable aspect and treating the event as commonsensical through her anaphoric use of the pronoun *es*/‘it’.

A similar instance is observable in Excerpt 3. Walter has just taken a baking tray with roasted paprikas from the oven and moves them into a saucepan with his bare hand, picking them up by the stem. Opposite from him, Zubir prepares a dough and they discuss whether and when Walter should taste it. In this moment, Walter picks up a paprika by its tip instead and burns himself.

Excerpt 3: STUDIO_BS_20190328_01.45.29

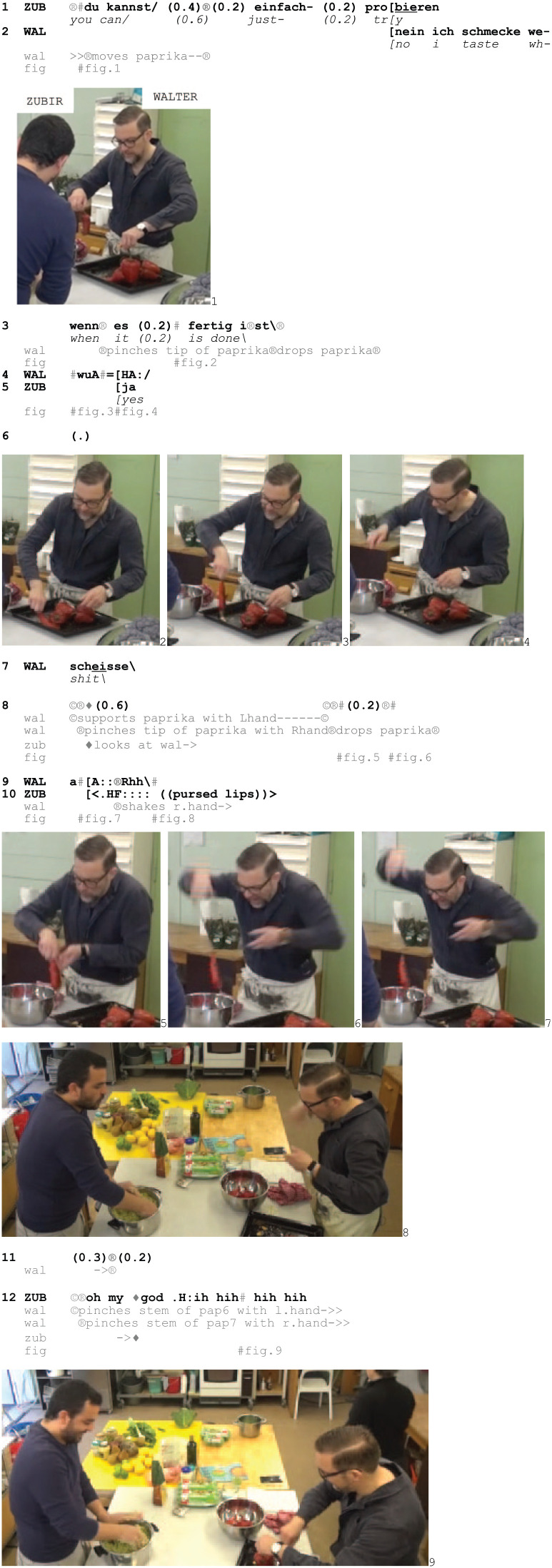

Walter is unproblematically moving several paprikas, pinching them by the stem (1, Fig. 1), before he takes a paprika by its tip (3, Fig. 2), drops it during his unfolding turn (3) and produces a vocal outcry with increasing pitch and loudness (4, Fig. 3–4) followed by *scheisse*/‘shit’ (7). Zubir treats this as making noticeable trouble as he looks up and monitors Walter (8) as he picks up the paprika again, now using both hands. Although Walter manages to move it, he drops it again (8, Fig. 5–6), and although his fingers no longer touch the paprika, Walter retracts his right hand and shakes it, while making another outcry (9, Fig. 7–8).

Zubir responds with an affiliative stance (cf. McArthur, 2021; Steensig, 2020; Stivers et al., 2011) through a hissing, loud inbreath with pursed lips, partly in overlap with Walter’s second outcry (10, Fig. 8) (cf. Keevallik et al., 2023) and the lexical *oh my god*, followed by laughter (12, Fig. 9). The use of this idiomatic expression in English further establishes the unfortunate character of the situation and the relevance to display sympathy.

Like Excerpt 1, Excerpts 2 and 3 show that members attend to burn displays as socially relevant. The sequential relationship between the practices that make the heat-occasioned sensorial experience noticeable and the responsive expressions is progressively established by the participants, and sometimes further elaborated through additional lexicalized accounts and complaints (see Excerpt 2).

The burn displays suspend the progressivity of both manual and conversational ongoing courses of action, which contributes to establishing the event as unexpected and ill-fated, which Goffman argues is instrumental in setting up the relevance to exhibit sympathy in response (Goffman, 1978: 804). It is notable that the persons burning themselves can extend (Excerpt 2) or repeat (Excerpt 3) the contact with the hot object, which indicates a certain relativity regarding what physiological suffering one can display that it is possible to endure. Furthermore, whereas Lea in Excerpt 1 orients to possible issues of blame by taking responsibility for why her peer burns herself on the tap water, the participants in Excerpts 2 and 3 construe the incident as a mishap. In this way they neither assume nor attribute responsibility for it (Pomerantz, 1978). This observation is relevant for subsequent sections where participants explicitly pursue issues of responsibility and inspect burn displays for their complainability and for what this makes socially relevant to do next.

### Orienting to issues of responsibility

The previous section showed that co-participants can respond to burn displays by treating them as unfortunate and unpredictable. This section highlights that co-participants can also sanction burn displays by treating them as something that could have been avoided if the other ‘had known better’. Instead of sympathy, this ‘occasions an attribution of responsibility for the “unhappy incident”’ (Pomerantz, 1978: 115).

In Excerpt 4, Amelia and Elena are preparing a pasta dish together when Elena grabs a spoon, which has been resting on the edge of a pot with boiling water and burns herself.

Excerpt 4: SCHOOL_HausW_SIS_20190430_I4_01.54.00

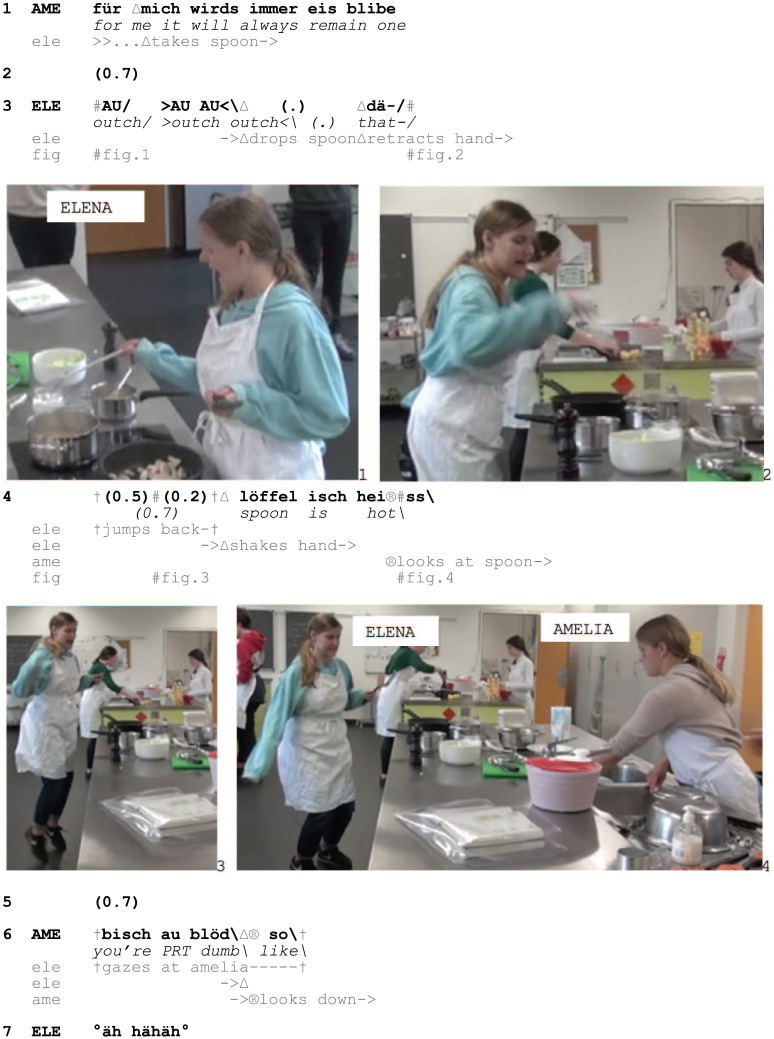

Soon after grasping the spoon (1) Elena makes her painful experience publicly available through grimaces and three lexicalized outcries (3, Fig. 1), before she drops the spoon, throws her hand into the air, and jumps backwards (3–4, Fig. 2, 3). Elena also initiates a turn with *dä-*/‘this-’ (3) but suspends it and only completes it with *löffel isch heiss*/‘spoon is hot’ after having jumped backwards and while shaking her hand (4, Fig. 3). In this way, the urgent character of the conduct is embodied in her movements but also in the suspension of her unfolding turn, and her verbal formulation that the spoon is hot.

During Elena’s turn Amelia looks at the spoon (4, Fig. 4), and then treats Elena’s conduct as blameworthy through *bisch au blöd*/‘you’re PRT dumb’ (6). The particle *au* ties the categorization *blöd* to Elena’s manipulation of the spoon, the high temperature of which is treated as self-evident. By making the common-sense nature of the material environment relevant for how she attends to the burn display, Amelia establishes the incident as anticipable and thus avoidable, and holds Elena responsible for it, insinuating that she should ‘know better’. Elena responds to this reproach with laughter, treating it as non-serious and mitigating it.

In Excerpt 5, we observe a similar procedure. Eva and Elis have been cooking together and now sit in the living room next to the kitchen. As Farid arrives and starts talking to Elis, Eva heads to the kitchen and takes some baguettes out of the oven with her bare hands. The excerpt begins as Eva takes out the second last piece of baguette and hastily drops it onto a cooling rack.

Excerpt 5: CookTrad_TUN_BL_20221016_52.25

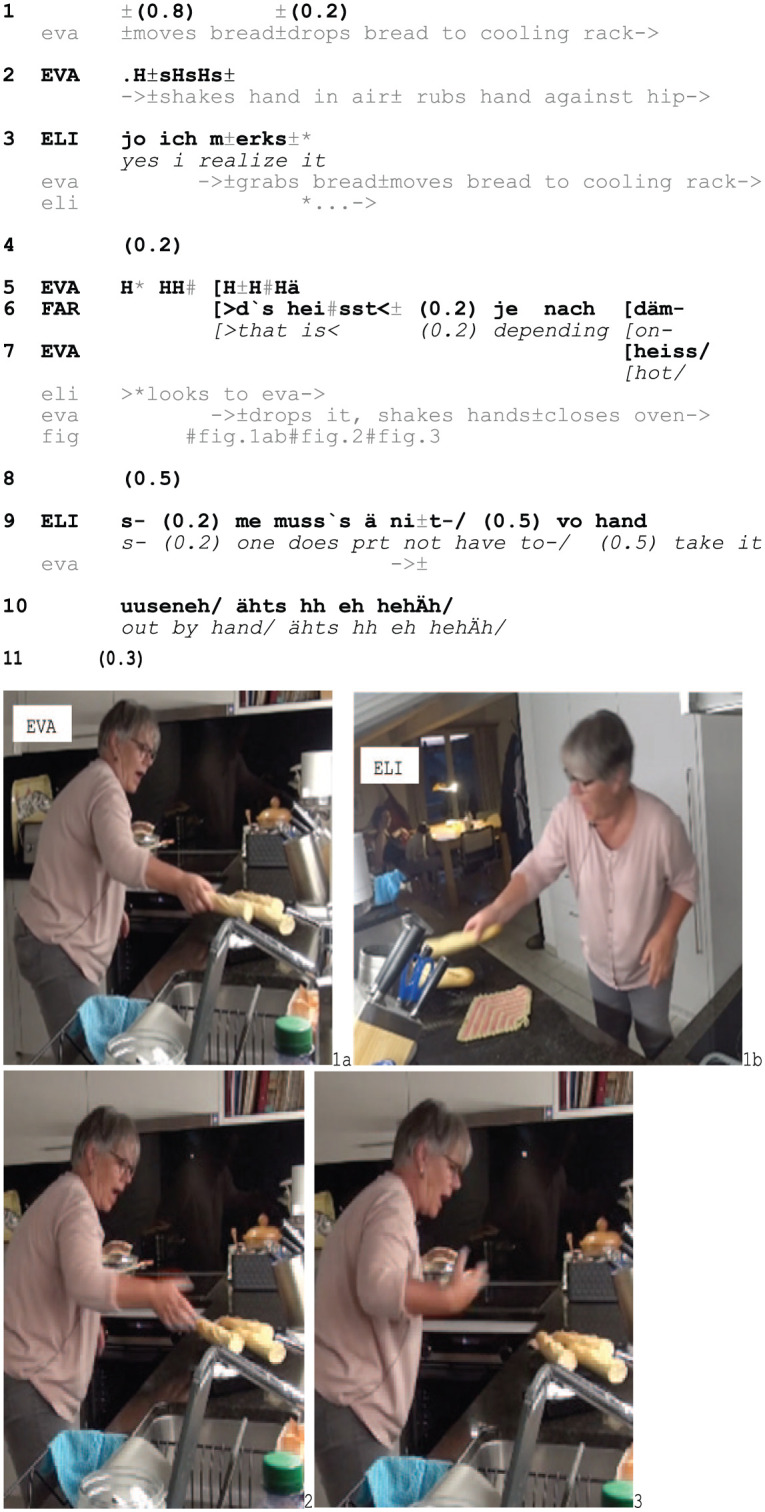

As Eva takes the third piece of baguette out of the oven she drops it (1) and breathes in hearably while cooling her hand in the air and against her hip (2). She then reaches for the last piece, repeating the procedure: producing a loud expiration, dropping the bread, and shaking her hand (3–6, Fig. 1–3, cf. Excerpt 2). She also grimaces (7, Fig. 1ab) and maintains this facial expression until accounting for her conduct through *heiss/*‘hot’ (7, Fig. 2–3), thereby displaying the sensorial experience as progressively painful. Similar to Excerpt 4, dropping the bread adds to the incident’s urgent character.

Elis orients to the audible features of Eva’s conduct, looking at her at the beginning of Eva’s expiration (5) before seeing her dropping the bread and grimacing (5–6, Fig. 1b). Shortly after (8), Elis sarcastically comments that it is not necessary to take the bread out of the oven with bare hands (9–10). The use of the particle *ä* (7, cf. *au* in Excerpt 4) ties this negative rule formulation to Eva’s handling of the baguette and the comment’s prosodic format and the laughter – which is not reciprocated – make it hearable as a criticism. In highlighting the avoidable character of the incident, Elis treats Eva’s conduct as related to commonsensical know-how regarding how to do things in the kitchen, which is used to frame the incident as being her own fault and the burn display to be blameworthy.

In Excerpt 6, matters of responsibility and self-evidence are also addressed in response to a burn display. Sam and his brother Farid cook together with Farid’s partner, Elis. Sam and Farid are frying *briques*, a spring roll-like dish from the Maghreb, when Farid gets burned by a splash of oil.

Excerpt 6: Cook_Birthday_BL_20200421_04.19.07

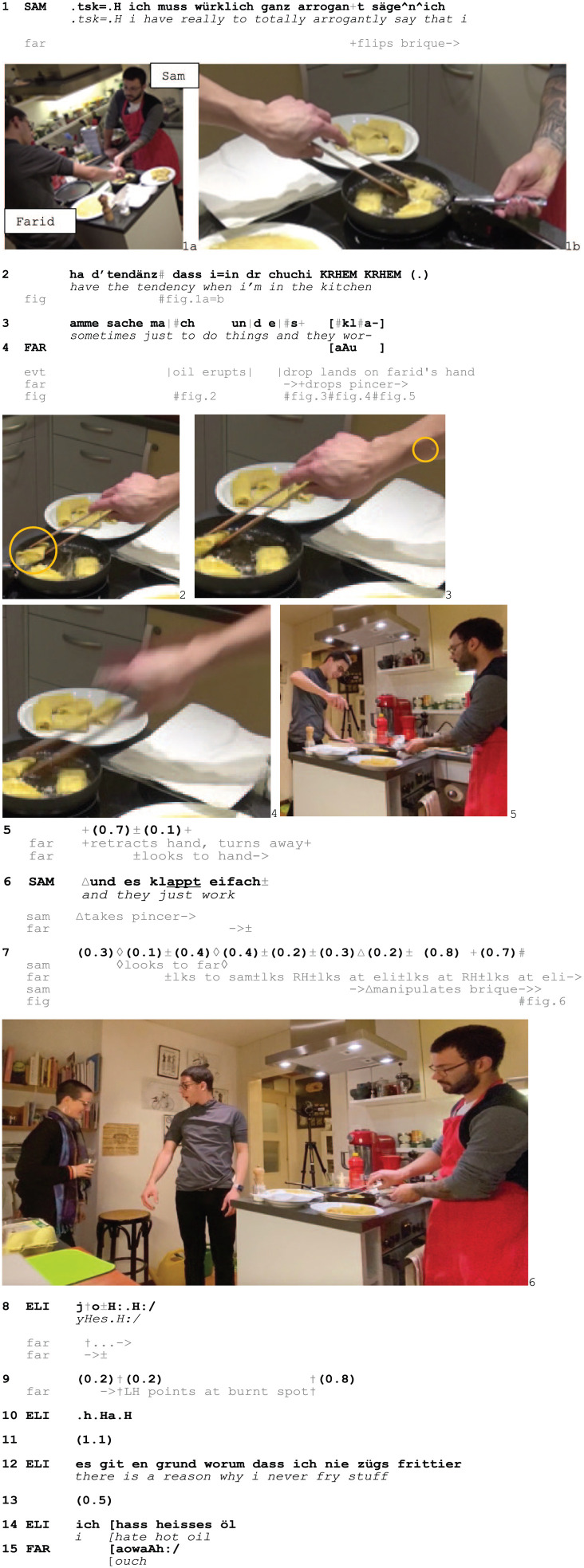

When the oil lands on Farid’s hand, he suspends the cooking by dropping the pincer, grimaces, retracts his arm (3, Fig. 2–5) and produces a pain cry (4). Sam treats this as socially relevant by suspending his own ongoing turn (3), and then orients to the relevance of assisting with the cooking, picking up the pincer (6) and continuing to fry the *briques* (7) – without further attending to the burn display. Farid then turns to Elis, alternatively looking at Elis and his arm (5–7) with an expression of stupefaction on his face (Fig. 6). Elis does not display sympathy either, downplaying Farid’s burn display by treating it as evident through the affirmative token *jo/*‘*yes*’ and by laughing (8). Farid understands this as resisting to affiliate with him (cf. Holt, 2010, 2012; Jefferson, 1979), as he pursues sympathy by pointing at the burnt spot (9). Elis however only responds with more laughter (10), further resisting to treat the burn display as a valid complaint. As Elis eventually accounts for her stance by stating that she would never engage with hot oil herself (12) and claiming her dislike of it (14), she invokes the predictable risk of getting burnt and maintains an attitude that Farid has himself to blame. In overlap, Farid produces another pain cry (15), further pursuing an affiliative response to his pain display.

In this section, we have seen how co-participants respond to burn displays by invoking issues of responsibility. This is achieved by treating the incidents as sequentially and socially implicative, but as something that could have been avoided if the other had ‘known better’. This shows that participants orient to the social accountability tied to such conduct: They resist to what the pain displays seem to make relevant in terms of affiliation and sympathy. Even in cases where co-participants assist with the ongoing activity in response to the burn (cf. Excerpt 6) and thus recognize the practical urgency of withdrawing from the heat source, they do not necessarily engage in conversational affiliative practices. This suggests that participants are sanctioned in case they complain about things that contradict what they ‘should’ know and that they monitor their respective undertakings for the practical and embodied know-how they exhibit.

### Questioning the validity of burn display

In the previous sections, we have observed that co-participants witnessing burn displays can orient to their unfortunate character but also to issues of responsibility, resisting to the social relevance of sympathy that a pain display occasions. In this section, we show that co-participants can question the validity of burn displays by engaging with the allegedly hot object themselves.

In Excerpt 7, Henri moves mashed potatoes from a cooking pot into a bowl while Simon, next to him, monitors the activity. Holding the spoon in his left hand, Henri attempts to use his right hand to stabilize the pot but retracts it several times, claiming that it is hot.

Excerpt 7: Heat_SCHOOL_HausW_DEL_20210122_K1_PM_ILOT2_01.12.49

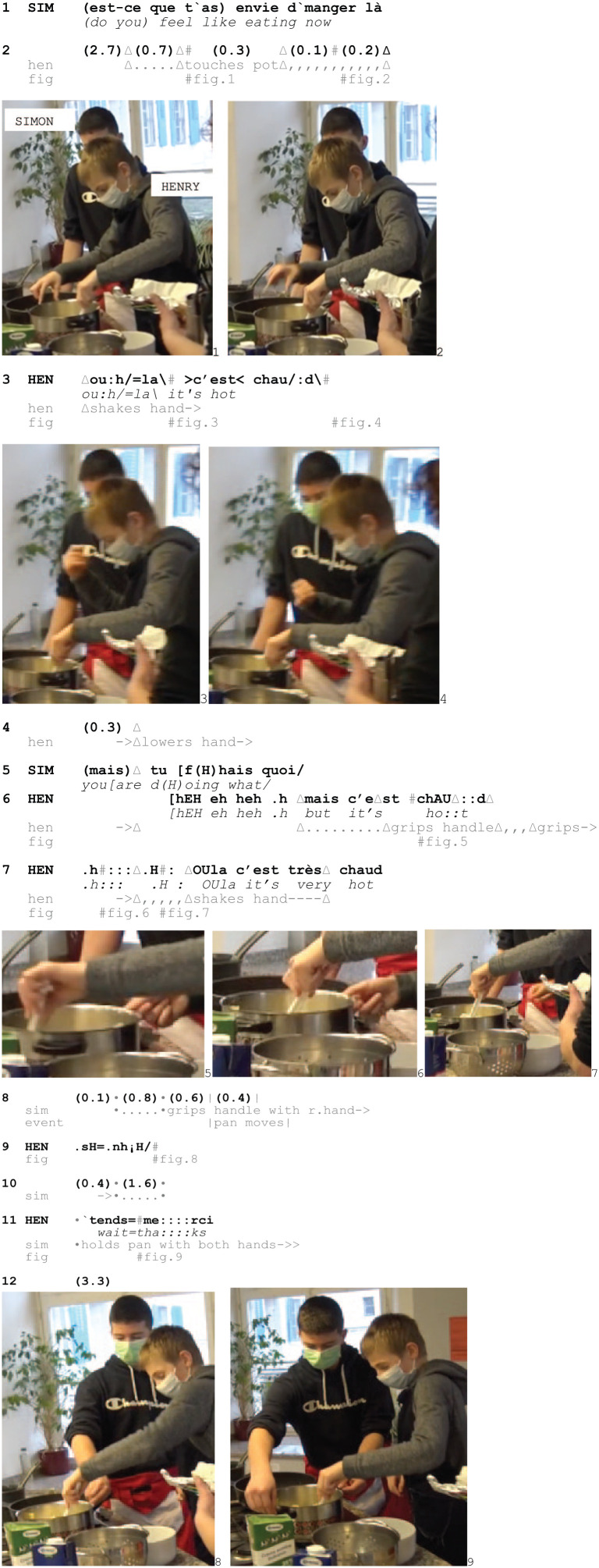

Instead of responding to Simon’s question (1), Henri focuses on the potato mash. His first attempt to hold the pot by gripping its side occasions a retraction of the hand (2, Fig. 1–2), which he transforms into a shaking that is accompanied by the outcry *ou:h/=la\* and the account that it is hot (3, Fig. 1–4). Simon questions what Henri is doing, with embedded laughter (5), which gives it a quality of jocular criticism. Henri hears it as targeting the legitimacy of his burn display and responds with laughter and a contrastive but-prefaced account (*mais c’est chau::d*/‘but it’s wa::rm’) (6), pushing against the criticism carried by Simon’s question. During his turn, Henri reaches for the pot a second time but as he grips the handle (6, Fig. 5), he lets it go again. He then grips it again before retracting his hand a third time, shaking it and producing the outcry *Oula* (7, Fig. 5) and a second, upgraded account (*c’est très chaud*/‘it is very hot’), insisting on the validity of his retraction.

In response, Simon extends his arm and grasps the pot’s handle (8–9, Fig. 8), visibly checking its temperature. He then stabilizes the pot with both hands by pushing down on the upper part of the handles (10–11, Fig. 9). Simon’s questioning of Henri’s conduct (5) and the subsequent checking of the pots’ temperature (8–9) challenge the legitimacy of Henri’s display that he is burning himself on the pot by accessing its experiential source. Moreover, the way Simon ends up holding the pot validates Henri’s displayed trouble, which is visible from *how* he holds it, and orients to Henri’s practical problem as socially implicative by providing assistance.

Thus, additionally to verbally questioning the validity of burn displays and the treatment of objects as being ‘too hot’, co-participants can establish a shared access to the experiential source of the burn display to verify whether the object is as hot as it is claimed to be. Furthermore, Simon’s access to the object occasions an alternative way of handling the material which both acknowledges Henri’s initial access as being painful and corrects *how* to access it. Simon’s way of holding down the pot shows an orientation to this as a practical issue of know-how concerning how to handle materiality – in this case, how to hold the pot without burning oneself.

In Excerpt 8, David begins to cut a pie after taking it from the oven, while Jamal, standing next to him, monitors the cutting activity (Fig. 1).

Excerpt 8: SCHOOL_HausW_DEL_20210129_K1_AM_ILOT3_01.43.00

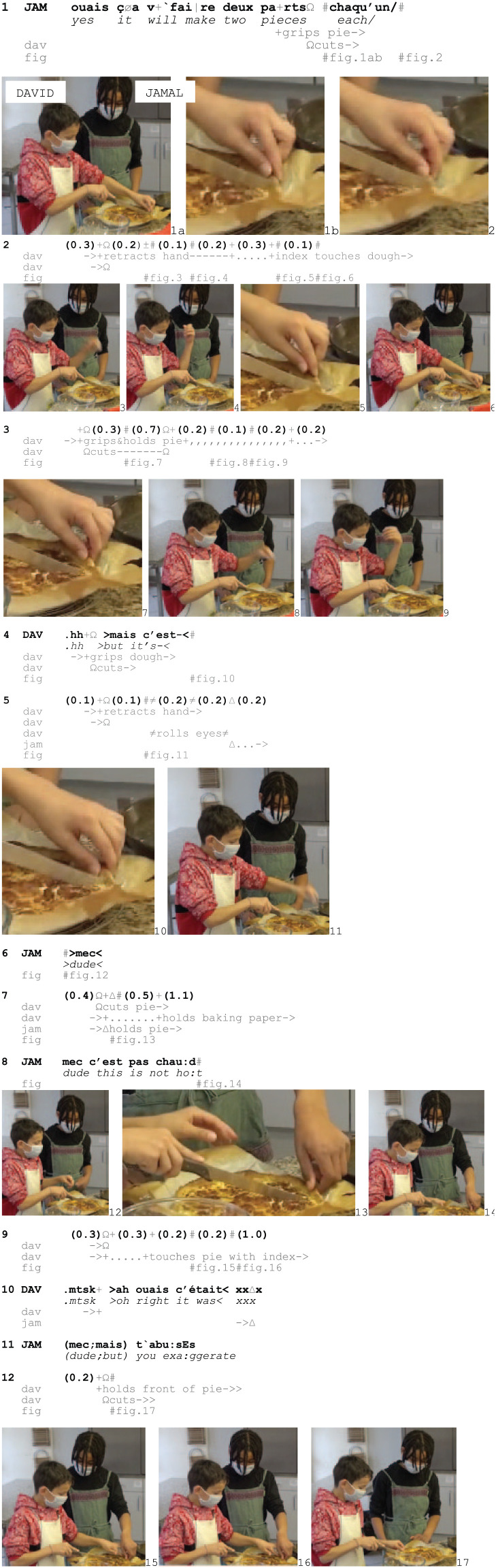

To keep the pie in place, David grips its crust (Fig. 1ab), presses it down and starts cutting (1, Fig. 1b–2). Shortly after, he suspends cutting by visibly minimizing haptic contact with the pie through a series of bodily retractions combined with voiced and unvoiced sounds.

After the first retraction (2, Fig. 3–4), David carefully checks the crust with his index finger (2–3, Fig. 5–6) before gripping it again (3, Fig. 7). However, he retracts his hand again (3, Fig. 8–9) and when gripping the pie for the third time (4, Fig. 10), he produces the uncompleted verbal turn *mais c’est-*/‘but it’s’, which in this sequential environment is hearable as projecting an adjective or a verb phrase referring to heat (like ‘but it’s warm’). The contrasting turn-initial *mais*/‘but’ establishes the unexpected character of the incident, accounts for suspending the cutting and shows David’s orientation to his own conduct as problematic.

As David retracts his hand for the third time (Fig. 11) and rolls his eyes, showing frustration (5), Jamal approaches his left hand (5–6, Fig. 12) and questions the validity of David’s conduct through the address term, *mec*/‘dude’ even before gripping the pie’s crust himself, thus establishing haptic contact with the object (6–7, Fig. 13, cf. Excerpt 7). Contrary to what David’s conduct implied, he then demonstrates that the pie is not that hot by holding it for more than 1.5 seconds (7, Fig. 13), before rebutting David’s claim verbally by repeating the address term *mec*, and negating David’s previously abandoned verbal turn through *c’est pas chaud*/‘it’s not hot’ (8, Fig. 14). By contesting the legitimacy of David’s burn display and criticizing his display of sensitivity in the face of what is treated as negligible degrees of pain (see Jenkins and Hepburn, 2015), Jamal exhibits his understanding of David’s conduct as being heat-occasioned while holding him accountable for delaying the cutting activity. David, in turn, verifies Jamal’s contesting claim by checking the temperature of the pie’s crust with his left index (9, Fig. 15–16), and verbally aligns with him through the change-of-state token *ah ouais*/‘oh right’ (10). This contributes to collectively establishing the pie as not *that* hot after all and validates Jamal’s accusation that David is exaggerating (11).

To be seen as exaggerating a behaviour, one must be seen as deliberately engaging in such behaviour, that is, by choice. Accusing David of exaggerating shows that displayed sensorial experiences can be treated as a deliberate social action and that members can question its validity. Like the paprikas in Excerpt 3 and the pot in Excerpt 6 the pie’s crust is treated as more or less hot depending on where and how one touches/holds it. Nevertheless, Jamal alludes to an issue of personal sensibility rather than to practical conditions. Finally, in response to Jamal’s allegations, David holds the pie in yet another place and only with two fingers (12, Fig. 17), which enables him to continue cutting. Thereby, he still treats the pie as hot and dismisses Jamal’s countering in an embodied way, despite his prior verbal alignment (cf. Excerpt 6).

This section has demonstrated that co-participants can question the validity of another person’s burn display. In those moments, the persons claiming to burn themselves are held accountable for their displayed experience, but also for suspending or interrupting the progressivity of initiated courses of action. In Excerpts 6 and 7 the participants progressively attend to the suspended course of action as embedded in their joint activity and orient to the practical and social implications of burn displays by assisting with the task – or not. Haptically accessing an object that another participant treats as too hot to touch treats the latter’s burn display as problematic and potentially sanctionable. This dimension notably surfaces in more or less overt allegations of exaggerating the displayed intensity of the sensorial experience, which reflexively establish the social accountability of such conduct and are indicative of its sequential implications: Even when co-participants treat the displayed intensity of heat-occasioned sensorial experiences as questionable or disproportionate by attending to the interactional noticing’s experiential source (Schegloff, 2007: 87 fn. 17; 219), they still orient to the burn displays as making relevant some further action.

### Treating a response to the burn display as noticeably absent

In the previous sections, we have seen that co-participants treat burn displays as making noticeable a sensorial experience and as sequentially and socially implicative. This section further corroborates our claim that participants treat burn displays as setting up an interactional relevance, by discussing a case where the person claiming to burn themselves treats an uptake by their peer as noticeably absent.

In Excerpt 9, Raphael has gone to fetch pre-heated plates for William and himself. While walking back to their workstation, Raphael loudly complains that the plates are hot and produces some embodied displays of pain.

Excerpt 9a: SCHOOL_HausW_DEL_20210129_K2_AM_ILOT1_01.58.07

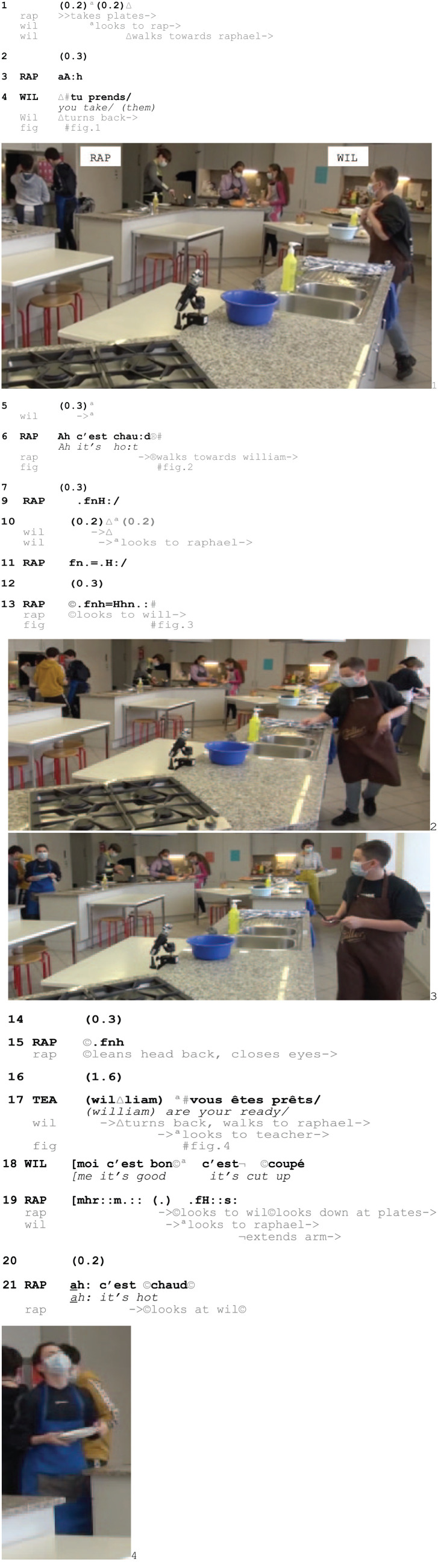

As Raphael takes the plates from the heating cabinet and walks back to the cooking station, he claims that they are hot through several audible vocal (3), verbal (6) and non-vocal (9) pain displays (Fig. 2–3). William monitors him continuously (1; 10; 18) and after engaging in mutual gaze with William (13, Fig. 3), Raphael produces more heavy breathings (13, Fig. 3). Furthermore, seeing that William sees him, Raphael repeats the loud inbreath and arranges a suffering posture by closing his eyes and tilting his head backwards while walking (15–19, Fig. 4). After upgrading the pain display further with vocalized sounds (19), he looks at William again, and seeing again that he is observed, he produces yet another hissing breathing (19) and verbal outcry, claiming that the plates are hot (21). Through the repeats, Raphael thus elaborates the burn display as recipient designed for his peer, and by coordinating the successively upgraded pain display with William’s gazes towards him, Raphael is seen as pursuing a responsive action from William, who resists to produce one. This shows that members displaying to experience pain monitor their co-participants’ (missing) uptake and can engage in pursuing this by ‘compos[ing] themselves and/or their environment to enhance the possibility of “noticing” by others’ (Schegloff, 2007: 87). Eventually, this results in an overt orientation to a response as missing:

Excerpt 9b: SCHOOL_HausW_DEL_20210129_K2_AM_ILOT1_01.58.07

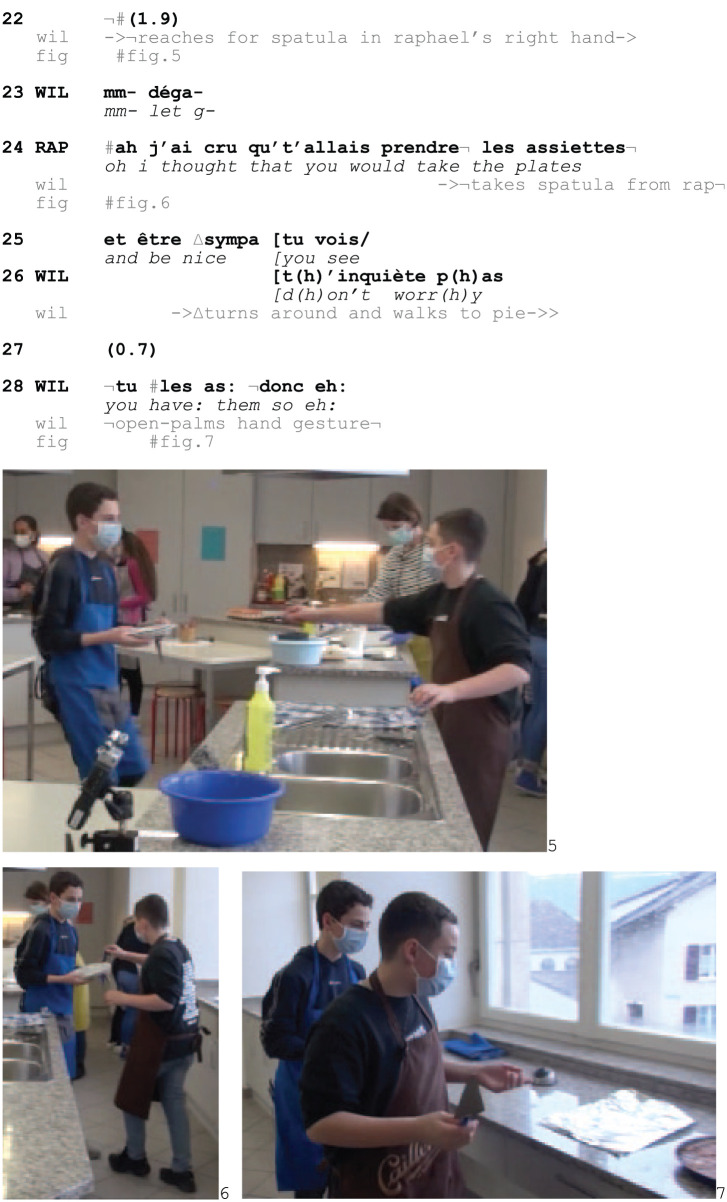

When William reaches for the spatula in Raphael’s hand (22–24, Fig. 5–6), Raphael complains about his expected response on behalf of William to be noticeably missing (24–25, Fig. 6) (Sacks, 1987; Schegloff and Sacks, 1973). The turn-initial *ah* indexes William’s request for the spatula as surprising, contrasting it with a formulation of what he thought William’s action trajectory was projecting: to assist with the plates. Furthermore, Raphael’s continuation points towards normative interactional stakes, treating what was expected (but didn’t happen) as the *sympa*/‘nice’ thing to do (25). Hence, Raphael both depicts an affiliation with his claimed suffering as noticeably absent, and formulates providing assistance as the relevant and expected thing to do. William dismisses this reproach by countering the practical reasoning behind the accusation, stating that the observable fact that Raphael is still holding the plates shows that there is no physiological urgency, thus invalidating Raphael’s claimed need for assistance. The dismissal is closed through an evidential hand gesture and William’s bodily disengagement from Raphael, as he turns around and focuses on the pie (28, Fig. 7).

This exchange shows that participants can retrospectively formulate and thus negotiate the rationale for how they understand and act on unfolding and prior events and whether this is justified or not. In this case, William is held accountable for not attending to and not assisting in response to a claimed burn display. The offered justification is that Raphael’s verbally claimed burn display is not proportionate to his embodied behaviour and therefore not credible – which, he claims, cancels the social relevance to assist him. This shows that members inspect displays of (painful) sensorial experiences for their authenticity and that this inspection, as a members’ analysis, is relevant for what they do next.

## Conclusion

This study shows the social accountability of burn displays. As practices for making noticeable heat-occasioned sensorial experiences, burn displays include (i) quick retractions from or rearrangements of the haptic contact with the hot object, (ii) verbal formulations of the problem, including accounts and complaints, (iii) audible out- and inbreaths and non-lexical vocalizations including outcries with distinct prosodic patterns. In several cases, the burn displays impede on the progressivity of the ongoing activity, as they are produced disjunctively instead of responding to a prior first action (see Excerpts 1, 6 and 7), which contributes to their urgent character.

Participants recurrently exhibit a practical dilemma regarding how to manage that pain impedes on ongoing activities by displaying to endure pain while pursuing their initiated course of manual action for some time. Meanwhile, the sudden suspension of an ongoing activity (as initiated and/or projectable bodily trajectories are interrupted and/or accelerated) is significant for how and whether burn displays are recognized as painful ‘incidents’. In Excerpt 9, William treats Raphael as *claiming* rather than *displaying* to burn himself, which, he reports, bears on a specific and inspectable bodily conduct: that Raphael sustains contact with the hot object (27). This shows that co-participants monitor whether burn displays are credible and proportionate to what they project as relevant next actions, which furthermore is consequential for whether and how they respond to them. Moreover, Raphael’s formulation of what was missing in William’s expected response as ‘being nice’ (24–25), shows that burn displays can be expectably bound to sequential and social implications – including offering help (cf. Excerpts 6 and 7). This can be tied back to the fact that burn displays are seen as connected to physiological and emotional experiences of pain. However, the practical possibilities for assisting depend on the object, the current participation framework, and the type of activity underway, showing the contingent and situated aspect of these incidents.

Co-participants recurrently attend to burn displays as unfortunate events (Excerpts 1–3) which, in turn, allows to develop complaints (cf. Schegloff, 2005: 460). However, participants inspect the complainability of displayed sensorial experiences, attending to whether it is proportionate to the expected thermic features of the object, and whether it is justifiable in relation to the implications it has for the ongoing course of action, that is, its suspension. Meanwhile, the absence of explicit affiliation in response to burn displays is itself revelatory, highlighting how participants orient to matters of responsibility as closely connected to trouble talk (cf. Pomerantz, 1978). Participants recurrently orient to issues of responsibility in response to pain displays, including by treating them as avoidable by pointing to pain as a common-sensical consequence of touching a known-to-be-hot object (Excerpts 4–6). Here, participants do not question the painful sensorial experience *per se* but treat it as one’s ‘own fault’, addressing the accountability of action. This finding suggests that participants orient to an expectation that one should show sympathy when someone is in pain, but not if the pain is self-inflicted. It also shows that members inspect practical activities for how they ought to be done and that participants may be held accountable for mishaps that could be prevented – including pain. This elaborates on research on pain displays in medical contexts (i.a. Heath, 1989, 1991; Weatherall et al., 2021).

Co-participants can also question the validity of burn displays, including by touching the object, occasioning defensive returns and accounts. Co-participants verifying the temperature by establishing a shared sensorial access to the heat source (Excerpts 7 and 8) can lead to assistance with the activity (Excerpt 7) or discarding the display as disproportionate (Excerpt 8). In this way, an object’s thermic features are reflexively established in situated, embodied ways in and through social interaction as an intersubjective achievement.

Reconsidering the visualization of the somatic reflex arc in Image 1 (p. 2) in the light of our analysis, shows that such conduct cannot be reduced to isolated, internal mechanisms. Rather, it is reflexively established through publicly available and emergently unfolding courses of action that develop its interactional relevance and social accountability. Although hand retractions – *when* they happen – are treated as consequential for the credibility of the claimed burn experience (see Excerpt 9), they co-occur with other multimodal resources within larger sequential environments and are thus performed as holistic phenomena. In this way, this study thus challenges atomistic approaches to bodily conduct.

This study also contributes to our understanding of retro-sequences (Schegloff, 2007) and the noticeability of events and conduct as interactional and locally assembled achievements that are designed and treated as being occasioned by perceptual (sensorial) incidents (Schegloff, 2007: 87, fn. 17) and ‘put on offer’ to be responded to (Schegloff, 2010: 47). In contrast with Goffman’s claim that response cries gain their *raison d’être* by virtue of their acceptability as ‘self-talk’, this study suggests that burn displays, as forms of ‘pain cries’, are treated as being produced for their witnessability by co-present members – which is instrumental for how they assemble their particular social accountability.

Finally, the results show that the question of what observable conduct is physiological and what is social is a members’ problem, including whether, how and to what extent the pain display is proportional to the object’s thermic features and the sequential and social implications that the burn display makes relevant. This contributes to research on how sensorial practices are made relevant in and for social interaction and shows how an EMCA approach to the study of sociality can be particularly productive for further investigating the intricate relation between physiological experiences and the social accountability of action.

## References

[bibr1-14614456251363736] Cekaite A Mondada L (eds) (2020) Touch in Social Interaction: Touch, Language, and Body. Routledge.

[bibr2-14614456251363736] Garfinkel H (1963) A conception of, and experiments with, ‘trust’ as a condition of stable concerted actions. In: Harvey OJ (ed.) Motivation and Social Interaction: Cognitive Approaches. Ronald Press, pp.87–238.

[bibr3-14614456251363736] Garfinkel H (1967) Studies in Ethnomethodology. Prentice-Hall.

[bibr4-14614456251363736] Garfinkel H Sacks H (1970) On formal structures of practical actions. In: McKinney JC Tiryakian EA (eds) Theoretical Sociology: Perspectives and Development. Appleton-Century-Crofts, pp.337–366.

[bibr5-14614456251363736] Goffman E (1978) Response cries. Language 54(4): 787–815.

[bibr6-14614456251363736] Goodwin MH (2017) Haptic sociality: The embodied interactive construction of intimacy through touch. In: Meyer C Streeck J Scott Jordan J (eds) Intercorporeality. Emerging Socialities in Interaction. Oxford University Press, pp.73–102.

[bibr7-14614456251363736] Haakana M (2001) Laughter as a patient’s ressource: Dealing with delicate aspects of medical interaction. Text 21(1–2): 187–219.

[bibr8-14614456251363736] Heath C (1989) Pain talk: The expression of suffering in the medical consultation. Social Psychology Quarterly 52(2): 113–125.

[bibr9-14614456251363736] Heath C (1991) The expression of pain and suffering in the medical consultation: Aspects of an interactional organisation. In: Conein B de Fornel M Quéré L (eds) Les Formes de la Conversation, vol. 2. CNET, pp.93–117.

[bibr10-14614456251363736] Holt E (2010) The last laugh: Shared laughter and topic termination. Journal of Pragmatics 42(6): 1513–1525.

[bibr11-14614456251363736] Holt E (2012) Using laugh responses to defuse complaints. Research on Language and Social Interaction 45(4): 430–448.

[bibr12-14614456251363736] ICOR Group (2007) Convention ICOR. Available at: http://icar.cnrs.fr/ecole_thematique/tranal_i/documents/Mosaic/ICAR_Conventions_ICOR.pdf (accessed 10 September 2025).

[bibr13-14614456251363736] Jefferson G (1979) A technique for inviting laughter and its subsequent acceptance/declination. In: Psathas G (ed.) Everyday Language: Studies in Ethnomethodology. Irvington Publishers, pp.79–96.

[bibr14-14614456251363736] Jefferson G (1984) On the organization of laughter in talk about troubles. In: Atkinson JM Heritage J (eds) Structures of Social Action. Cambridge University Press, pp.347–369.

[bibr15-14614456251363736] Jefferson G (2004) Glossary of transcript symbols with an introduction. In: Lerner GH (ed.) Conversation Analysis: Studies from the First Generation. Benjamins, pp.13–31.

[bibr16-14614456251363736] Jenkins L Hepburn A (2015) Children’s sensations as interactional phenomena: A conversation analysis of children’s expressions of pain and discomfort. Qualitative Research in Psychology 12(4): 472–491.

[bibr17-14614456251363736] Keevallik L Ogden R (2020) Sounds on the margins of language at the heart of interaction. ROLSI 53(1): 1–18.

[bibr18-14614456251363736] Keevallik L Hofstetter E Weatherall A , et al. (2023) Sounding others’ sensations in interaction. Discourse Processes 60(1): 73–91.

[bibr19-14614456251363736] Kreplak Y Mondémé C (2014) Artworks as touchable objects. Guiding perception in a museum tour for blind people. In: Nevile M Haddington P Heinemann T , et al. (eds) Interacting with Objects. Benjamins, pp.295–318.

[bibr20-14614456251363736] La J Weatherall A (2020) Pain displays as embodied activity in medical interactions. In: Wiggins S Cromdal KO (eds.), Discursive Psychology and Embodiment: Beyond Subject-Object Binaries. Palgrave Macmillan/Springer Nature, pp.197–220.

[bibr21-14614456251363736] Lindwall O Mondada L (2025) Sequence organization in the instruction of embodied activities. Language and Communication 100(1): 11–24.

[bibr22-14614456251363736] McArthur A (2021) Pain displays as a resource for action. In: Robles JS Weatherall A (eds) How Emotions Are Made in Talk. Benjamins, pp.263–286.

[bibr23-14614456251363736] Moerman M Sacks H (1988) On “understanding” in the analysis of natural conversation. In: Moerman M (ed.) Talking Culture: Ethnography and Conversation Analysis. University of Pennsylvania Press, pp.180–186.

[bibr24-14614456251363736] Mondada L (2018) Multiple temporalities of language and body in interaction: Challenges for transcribing multimodality. ROLSI 51(1): 85–106.

[bibr25-14614456251363736] Mondada L (2020) Audible sniffs: Smelling-in-interaction. ROLSI 53(1): 140–163.

[bibr26-14614456251363736] Mondada L (2021) Sensing in Social Interaction: The Taste for Cheese in Gourmet Shops. Cambridge University Press.

[bibr27-14614456251363736] Mondada L Bouaouina SA Camus L , et al. (2021) The local and filmed accountability of sensorial practices: The intersubjectivity of touch as an interactional achievement. Social Interaction: Video-Based Studies of Human Sociality 4(3): 128160.

[bibr28-14614456251363736] Nishizaka A (2020) Multi-sensory perception during palpation in Japanese midwifery practice. Social Interaction: Video-Based Studies of Human Sociality 3(1): 120256.

[bibr29-14614456251363736] Pomerantz A (1978) Attributions of responsibility: Blamings. Sociology 12(1): 115–121.

[bibr30-14614456251363736] Robinson JD (ed.) (2016) Accountability in Social Interaction. Oxford University Press.

[bibr31-14614456251363736] Sacks H (1987) On the preferences for agreement and contiguity in sequences in conversation. In: Button G Lee JRE (eds) Talk and Social Organisation. Multilingual Matters, pp.54–69.

[bibr32-14614456251363736] Sacks H (1992) Lectures on Conversation, vols. 1 and 2 ( Jefferson G , ed.). Blackwell.

[bibr33-14614456251363736] Sacks H Schegloff EA Jefferson G (1974) A simplest systematics for the organization of turn-taking for conversation. Language 50(4): 696–735.

[bibr34-14614456251363736] Schegloff EA (1992) Repair after next turn: The last structurally provided defense of intersubjectivity in conversation. American Journal of Sociology 97(5): 1295–1345.

[bibr35-14614456251363736] Schegloff EA (1996) Confirming allusions: Toward an empirical account of action. American Journal of Sociology 102(1): 161–216.

[bibr36-14614456251363736] Schegloff EA (2005) On complainability. Social Problems 52(4): 449–476.

[bibr37-14614456251363736] Schegloff EA (2007) Sequence Organization in Interaction. Cambridge University Press.

[bibr38-14614456251363736] Schegloff EA (2010) Commentary on Stivers and Rossano: Mobilizing response. ROLSI 43(1): 38–48.

[bibr39-14614456251363736] Schegloff EA Sacks H (1973) Opening up closings. Semiotica 8(4): 289–327.

[bibr40-14614456251363736] Song L Licoppe C (2024) Noticing-based actions and the pragmatics of attention in expository live streams. Noticing ‘effervescence’ and noticing-based sequences. Journal of Pragmatics 226(1): 1–16.

[bibr41-14614456251363736] Steensig J (2020) Conversation analysis and affiliation and alignment. In: Chapelle C (ed.) The Concise Encyclopedia of Applied Linguistics. Wiley Blackwell, pp.944–948.

[bibr42-14614456251363736] Stivers T Mondada L Steensig J (eds) (2011) The Morality of Knowledge in Conversation. Cambridge University Press.

[bibr43-14614456251363736] Stukenbrock A (2023) Temporality and the cooperative infrastructure of human communication: Noticings to delay and to accelerate onward movement in mobile interaction. Language and Communication (92): 33–54.

[bibr44-14614456251363736] Vatanen A (2023) Embodied noticings as repair initiation: On multiactivity in choir rehearsals. In: Haddington P Eilittä T Kamunen A , et al. (eds) Complexity of Interaction: Studies in Multimodal Conversation Analysis. Palgrave Macmillan, pp.99–141.

[bibr45-14614456251363736] Weatherall A Keevallik L La J , et al. (2021) The multimodality and temporatlity of pain displays. Language and Communication (80): 56–70.

